# “To be, or not to be”: experiencing deterioration among people with young-onset dementia living alone

**DOI:** 10.1080/17482631.2018.1490620

**Published:** 2018-07-05

**Authors:** Aud Johannessen, Knut Engedal, Per Kristian Haugen, Marcia Cristina Nascimento Dourado, Kirsten Thorsen

**Affiliations:** a Norwegian National Advisory Unit on Ageing and Health, Vestfold Hospital Trust, Tønsberg, Norway; b University of South-Eastern, Vestfold, Norway; c Center for Alzheimer’s Disease and Related Disorders, Institute of Psychiatry, Universidade Federal do Rio de Janeiro, Rio de Janeiro, Brazil; d Oslo Metropolitan University, Oslo, Norway

**Keywords:** Coping, existential needs, health care, health promotion, longitudinal qualitative study, subjective experiences, young people

## Abstract

Having dementia before the age of 65 (YOD) represents a radical break from an age-normative and expected life course. The disease afflicts the person’s identity, threatens the self-image and self-confidence, and erodes the person’s plans. The aim of the study was toexamine how people living alone with YOD perceive the course of dementia, their needs, and coping strategies, with a focus on narrating everyday life experiences. A longitudinal study using a qualitative approach was used. Five interviews, each with 10 informants, took place every 6 months from 2014 to 2017. The main theme is the person’s experiences of changes of identity over time. The most significant aspects of their experiences of the dementia affecting them and their reactions are these: the initial signs, coping efforts, concealing the diagnosis, social retraction, existential anxiety, revival of the self, worse and worse, and health personnel as background. The study concluded thatpeople with dementia are able to describe their experiences and needs for a long time during the progression of dementia. Their voices should be listened to for planning of services. Personalized care should be used to support them in order to preserve their identity in a normalized everyday life as far as possible.

## Introduction

Living with dementia before the age of 65 represents a break with an age-normative and expected life course. The disorder has mostly been conceived as a disease that older people suffer from (Prince et al., 2013), but younger people may also have a dementia disorder and are often referred to as people with young-onset dementia (YOD). Compared to the calculated 78,000 people above the age of 65 years with dementia in Norway, the number of people with YOD in Norway is estimated to be 4,500–5,000 (Engedal & Laks, 2017; Prince et al., 2013; Zhu et al., 2015).

The diagnosis of the progressive degenerative disease of dementia is a dramatic message to receive. It implies a reorientation of life and prospects for a “normal” ageing. The disease inflicts the person’s identity, threatens their self-image and self-confidence, and erodes plans for the future. Cognition will gradually decline among people with dementia (Harvey, Skelton-Robinson, & Rossor, 2003; Koedam et al., 2008; Luscombe, Brodaty, & Freeth, 1998), and the abilities to take care of daily activities and themselves, and to localize and move around on their own will deteriorate. The development of YOD and receiving the dementia diagnosis are reasonably followed by anxiety, turmoil, depression (Haugen, 2012; Johannessen & Möller, 2013), reduced quality of life and increased problems in everyday life in relation to work, friends and family (Spreadbury & Kipps, 2017). Behaviour will change, and behavioural changes are more frequent in YOD than in people with late-onset dementia (LOD; van Vliet et al., 2012). Usually, people with YOD are diagnosed at a later stage of the disease compared to people with LOD (Harvey et al., 2003; Koedam et al., 2008; Luscombe et al., 1998). In Norway, studies indicate that about 50% of people with dementia who receive homecare services have not been diagnosed with dementia. The same applies to about 20–30% of people with dementia living in a nursing home (Selbaek, Kirkevold, & Engedal, 2007). However, not everyone with possible dementia wishes to participate in a diagnostic process.

In addition, in research on dementia, there has been less attention given to how people with dementia experience and evaluate their situation, and how the environment can support their resources and abilities. Also, people with dementia actively respond and adapt to the disease with their resources and abilities (Johannessen & Möller, 2013; Spreadbury & Kipps, 2017).

Still, the essence of the experience of dementia is that the development of self is eroded (Caddell & Clare, 2010; Mograbi, Brown, & Morris, 2009; Westius, Kallenberg, & Norberg, 2010). The self can no longer be maintained and linked to memory and recollection of the past, of the autobiography; and the cognitive ability to connect events, places and people becomes lost (Jetten, Haslam, Pugliese, Tonks, & Haslam, 2010). Gradually, the knowledge of who “I was” and whom “I developed into” weakens. The context of self-recognition is fragmented, the external continuity in work and family relations weakens, and close relationships change, worsen or break down. Many people with dementia experience increasing isolation and loneliness (Johannessen & Möller, 2013). The loss of the functional ability to orientate oneself in and master one’s existence may give an experience of the loss of dignity and a sense of shame and despair (Norberg, 2017; Tranvåg, 2017).

According to the World Health Organization and government policies in Norway, people with dementia should remain living at home for as long as possible (Helse-og omsorgsdepartementet. (HOD)., 2015; WHO, 2012). However, a review study shows that people with dementia living alone have an increased risk of moving to a nursing home compared to those living with a partner (Luppa et al., 2009). Additionally, people living alone with dementia have significantly more unmet needs (Miranda-Castillo, Woods, & Orrell, 2010). People with YOD need support during the whole course of the disease (Harris & Keady, 2009; Horndalsveen, 2017; Roach, Keady, Bee, & Hope, 2009), yet few services supporting YOD have been established (Chemali, Schamber, Tarbi, Acar, & Avila-Urizar, 2012; Gjøra, Eek, & Kirkevold, 2015).

Studies report that health personnel’s evaluation of what people with YOD need is not necessarily in accordance with their subjective needs (Chemali et al., 2012; Frank & Forbes, 2017; Haugen, 2012). It is important for people with dementia to be given opportunities to express their thoughts, feelings and perceptions of their present needs and situation (Rostad, Hellsen, & Enmarker, 2013; Johannessen & Möller, 2013; Spreadbury & Kipps, 2017; Svanström & Sundler, 2015). Furthermore, it is well documented that people with dementia can express their lived experiences of having dementia, their perceived coping strategies and needs (Frank & Forbes, 2017; Hunderi & Hunderi, 2009; Roach et al., 2009; Willis, Chan, Murray, Matthews, & Banerjee, 2009). However, studies are scarce on how people with YOD experience the deterioration of dementia when living alone over time and how their existence and everyday life are affected. Therefore, we have performed this longitudinal study aiming to explore the existential experiences and coping of people with YOD, as they narrate the deterioration due to dementia that they go through over time.

## Methods

### Design

The study is a longitudinal explorative, descriptive study with a duration of 2 years. Individual qualitative interviews were conducted five times. The first took place at the inclusion (2014), and the other four interviews took place every 6 months thereafter. This rather short period between the interviews was considered appropriate, since significant aspects of dementia and the life situation may change rapidly, such as needs for services or moving into a nursing home. All interviews covered everything that the people had experienced on the day of the interview, as well as earlier experiences and reactions.

### Participants

To attain heterogeneity, we included people with YOD (age at onset of dementia before 65 years of age) of both genders and living alone, from the southern and western parts of Norway. They were recruited from six memory clinics. The informants could have different dementia diagnoses and various co-morbidities. However, frontotemporal dementia was an exclusion criterion, because people with this condition are less aware that something wrong. A total of 10 people with YOD were asked to participate, and none declined. Thus, the sample at the inclusion time (at the first interview) consisted of 10 informants, aged 49 to 67 years of age (mean 60 years), including 7 women and 3 men. One participant dropped out after the first interview, and after the third interview 1 person dropped out and 1 person died, which resulted in 42 interviews in all. Regarding the person aged 67 years, the symptoms had started many years before the study (unknown how many but before the age of 65 years), and this informant is therefore included in the sample. Eight people were divorced, one was a widow, one was unmarried. Among the 10 informants, 3 were childless, 4 had a daughter, 1 had 2 daughters, 1 had a son and a daughter, and 1 informant had 2 sons—altogether 10 children. Four informants had (six) children living in other parts of the country; thus, only three of them had children living at the same place, but not in the same household. Other characteristics of the informants are described in Table I. The participants might be in somewhat different stages of dementia, even if the diagnoses of all were set within six to eight months before the first interviews took place.

**Table I.** Overview of the first interview—the other four interviews took place every 6 months)—and the characteristics of the informants.

Gender(age)	1. Interview: residence/services (min) ^a^	2. Interview: residence/services (min) ^a^	3. Interview: residence/services (min) ^a^	4. Interview: residence/services (min) ^a^	5. Interview: residence/services (min) ^a^
W (52)	Nursing home/support contact ^b, f^ (57)	Home/support-contact (40)	Home/support contact (37)	Nursing home/support contact (28)	Nursing home/support-contact (40)
W (67)	Home/house cleaning ^d, e^ (40)	Nursing home/support-contact (31)	Nursing home/support-contact (18)	Nursing home/support-contact (19)	Nursing home/support-contact (18)
W (59)	Home/house cleaning ^e^ (42)	Home/house cleaning ^d, e^ (16)	Home/house cleaning ^d, e^ (38)	Home/house cleaning ^d, e^ (22)	Home/house cleaning ^d, e^ (26)
W (57)	Home (31)	Dropped out			
W (60)	Home/daycare centre x 2 ^d, e^ (30)	Home/daycare centre x 3 ^d, e^ (17)	Home/daycare centre x 3 ^d, e^ (23)	Home/daycare centre x 3 ^d, e^ (21)	Home/daycarecentre x 3 ^d, e^ (19)
W (64)	Home/house cleaning/support-contact ^d, e^ (41)	Home/house cleaning/support-contact ^d, e^ (29)	Home/house cleaning/support-contact ^d, e^ (42)	Dropped out	
W (49)	Home/daycare centre x 1 ^e^ (55)	Home/support-contact ^e^ (43)	Home/support-contact ^e^ (63)	Home/support-contact ^d, e^ (40)	Home/support-contact ^d, e^ (56)
M (65)	Home ^c^ (29)	Home ^c, d^ (25)	Nursing home ^d, e^ (22)	Nursing home ^d, e^ (17)	Nursing home ^d, e^ (36)
M (61)	Home/activity group x 1 ^c^ (22)	Home/activity group ^c^ (19)	Home/daycare centre x 3 ^e^ (19)	Dropped out	
M (64)	Home ^c^ (25)	Home ^c^ (15)	Home ^c^ (18)	Home ^c^ (13)	Home ^c^ (25)

^a^Length of the interview. ^b^A support-contact is a person (employed by the municipality) that helps another person with social contacts and activities. ^c^Still driving. ^d^Help with economic issues from family. ^e^Support with medication administration. ^f^Living in a nursing home shortly before moving to her own flat.

### The interviews

The 42 interviews lasted for 13 to 63 minutes each, and the interview time came to a total of 1267 minutes (mean 25 minutes) (see Table I). The interviews, conducted by the first author (A.J.), were performed at the most convenient place for the informants (Denzin & Lincoln, 2011; Kvale & Brinkmann, 2010). Two informants had their first interviews at a hospital. All the other interviews were carried out in the informant’s private home or in a nursing home (11 interviews). All interviews were tape-recorded and transcribed verbatim by a professional typist within 2 weeks. A quality control check was performed by interviewer A.J., who listened to the tapes while reading the interviews. The questions are listed in Table II. The first question in the interview guide was asked only at the first interview. Depending on the replies, the aspects and ideas raised by the informants led to further questions to obtain additional information. Detailed follow-up questions explored more specifically what had happened since the last interview. The informants were posed a summarizing open question: “All in all, how have you experienced the changes in your situation since we talked together the last time?”

**Table II.** Questions and themes in the interviews of the people with young-onset dementia living alone.

Can you describe how it all started, the changes, and the process of being diagnosed (only in the first interview)?
How is your everyday life after you received the dementia diagnosis?
Can you describe the changes you have experienced and how you cope with them?
Does the disorder affect your relationship or contact with family members or other people?
Do you feel that you are included in the treatment and services you receive?
Have you experienced other people taking a decision for you? If yes, how does that make you feel?
Has your quality of life changed after receiving the dementia diagnosis?
Have you the need for support or information of any kind?

### Analysis

Corbin and Strauss (2008) have outlined a reformulated approach to grounded theory. The modified method is particularly fruitful for the study of people living experiences of personal development and social interactions, making it the preferred method for this longitudinal study. The method by Corbin and Strauss (2008) was applied, without the intention of formulating a theory, but to acquire more detailed knowledge about how people with YOD experience their life situation and their needs for support in everyday life. In line with this method, the transcribed interviews were analysed with a focus on people with YOD living alone, their experiences and their efforts to cope with the deleterious effects of dementia over time. The analysis was performed in line with the modified grounded theory version of analysis (Corbin & Strauss, 2008) by two of the authors (A.J. and K.T.). At first, the initial “open coding” was to read all the interviews open-mindedly whilst watching for relevant themes. Then the themes were compared by “axial coding”, to find the most relevant higher-level themes for the whole group. By this process, sub-themes and variations were formed. Agreement on higher-level themes was reached by discussion among the researchers. In the process, the researchers continuously related the themes to the empirical material, analysing it *vertically*, and going back and forth in the material of one informant. We also analysed it *horizontally*, comparing the informant’s situations and experiences of all the informants, in line with the method’s constant comparative approach. The process of open and axial coding, as Corbin and Strauss emphasize (2008, p. 198), are intertwined empirically. The axis of the empirical analysis is the time perspective, how the dementia process changed their life situation, as narrated during the course of the first and the four follow-up interviews. We examined the informants’ experiences, challenges, strategies and support, as they developed over time.

### Ethics

The study followed the ethics outlined in the revised Declaration of Helsinki (World Medical Association,2013) and was approved by the Regional Committee for Ethics in Medical Research, Southern Norway (number 2013/2149). The Norwegian Data Protection Authority also approved the study (number 36,797). The informants received oral and written information about the study and gave their written consent before they were interviewed.

## Findings

The narratives’ styles and the content varied greatly. Some are rather simple stories about everyday living, concentrating on events, activities and daily routines. They are matter-of-fact stories, containing few reflections and emotions, and seem to represent the informant’s habitual narrative style. The emotions are subdued, implied in a few words and remarks. The last stories these informants tell are usually much like the first, with small changes; the forgetfulness has increased somewhat, there are more social retractions and some activities have ended. But their dominant feelings and moods, and prominent coping styles, seem to be mostly preserved during the 2-year period.

### Changes of identity over time

The main overriding analytic line in the empirical presentation is *time*, and the main overriding existential theme is *changes of identity over time*. The eight *themes* condensing the most significant aspects of their informants’ experiences of how dementia is influencing them are: the initial signs, coping efforts, concealing the diagnosis, social retraction, existential anxiety, revival of the self, worse and worse, and health personnel as background. Time is represented by the succession of the themes as well as within the themes. A few of the informants have experienced all the themes, others only some of them.

### The initial signs

The informants’ onset of dementia was marked by various signs of memory problems, from a rather imperceptible increase in forgetfulness going on for years—forgetting names, how to handle things, having problems with orientation and localization. Increasing memory problems and compensation efforts gradually interfered with everyday functioning. For several people, difficulties with performing tasks were observed at the work place. Except for one, all were working when the first signs started. A visit to the doctor was recommended, and the assessment process started. The procedures, tests and assessments for the diagnosis are mentioned as stressful for some, a rather brutal disclosure of personal incompetence. A woman said:

“With all the tests before the diagnosis was set, emotionally I fell down. There was great despair when I did not manage even simple tasks, quite elementary things.”

The diagnosis and the implications were experienced by all of them as dramatic, in one way or another. It was a message abruptly turning upside-down their total existence, their everyday life and their plans for the future. Another woman narrated:

“My tears just ran and ran. That was good for me. It was sorrow. In that way, I released some of it.”

As she described, crying was a start to absorbing her shock and beginning the process of adjusting and coping.

The interviewees reported problems with localization, even at well-known places like the shopping mall. Orientation in space is lost, as one reported:

“I have to sit down and think: Where was this, where am I? Things that went automatically earlier.”

During the diagnostic process, the nine people still working were proposed a full- or part-time sick leave or retirement. This was a significant and decisive turning point in their life’s trajectory, the start of social retraction. One person was still working (but on sick leave) at the first interview. She recounted that more advanced work tasks were taken away from her: “I have felt very left out at work. Degraded and you feel the ground slipping away.” She still did not believe in the dementia diagnosis: “I feel I function so well in many other ways. I do not feel I am ill.” Her work represented a central part of her identity. Some experience the unexpected loss of work with anguish, as a blow to their main self, like this retired man:

“To have to retire is terrible, but I manage it. It is too early, but I will not be a clown at my job.”

### Coping efforts

Because of reduced memory functions, all the informants were using the coping strategy of taking notes—for appointments, things to buy, visits. A woman told us: “I live in such a yellow Post-it system.” Another woman called herself “a note woman”. As the memory problems increased, they all tried to compensate by taking even more notes. Emotional, cognitive and social reactions interacted. For some, the dementia disorder also implied disturbed and uncontrollable emotional reactions. A woman related how she was no longer able to control her emotions: “I can’t control it, can’t do anything about it. It just pours out.”. She explained further:

The dementia disorder has changed my brain in the way that I am no longer as concentrated, and I often forget, but I have also emotionally changed a lot. I cry very easily. It is terrible. I am so ashamed. I can laugh at the wrong place, and later find out that it was misplaced, because other people send me strange looks.

The varied symptoms of dementia destroy social interaction, reduce social contact, and break social norms producing shame and anxiety. Isolation breeds depression: “I became depressed, because I felt so left out.” The existential threat of dementia is mastered by concentrating on *living in the present*. The informants presented this as their main adaptive cognitive and emotional coping strategy, especially in the earlier stages: “I think it is very wise not to think about tomorrow. That is stressful.” Another said: “It is of no use to protest. You just have to take it and enjoy the good days.” “To get the best out of it, live one day at the time” summarizes their constructive adaptation. How they manage it varies.

To be a person in good spirits was mentioned as an asset. A man emphasized:

“I am a person who always look at the bright side of life—that it is more good than bad. Then I function well, and I do not go and pity myself.”

To have strong self-confidence gives resilience and protects the self. He continued: “I still believe in myself, so I say: ‘This is a disease, and not my fault. It is just the way it is.’ So, I think this will be OK, if it does not get worse.” A woman compared her condition with others, and thus normalized it: “It is like a normal life. Sometimes I find it a little boring; other times it is OK. I think it is like this for most people. And I do not have pain.” Reaching a higher age implies a realization that other elderly people also have diseases and problems.

### Concealing the diagnosis

All informants, except one, mentioned that they have controlled their outer image by concealing the diagnosis; they have presented their difficulties as a “memory problem”. Their reasons are to be seen as “normal”, to be able to relate to others as usual, and diminish the impact of the disease—and also for themselves: “I want to live my life as well as possible, and not talk very much about it. Yes, not enlarge it by talking.” In the early stages, their problems may be minor, and some disguised them with jokes and self-irony. A man mentioned his disease as “a touch of dementia”. He commented: “I do not talk much about illness in general. I think I may get more ill by talking.” Another man informed us: “I have not used the word *disease* yet.” His reason for not telling others was “that *then* people will believe that it is much worse than it is”. In such a way they protect their image, and self-image.

Looking healthy makes concealment easier: “I am healthy outside and ill inside.” Being and looking rather young, they have misled others into not believing in a diagnosis of dementia. One informant said:

“When you change [in this way] at my age, I experience that nobody understands it. Not at all. They are all concerned that I should pull myself together, and that is impossible.”

She has told only her former husband and her daughter about the diagnosis:

“They are just angry at me, they do not understand this.”

The informants feel that dementia is associated with old people and is a taboo. A woman working part-time with a highly skilled job, said:

“It is very taboo for me. For others, also—I feel. If you have got Alzheimer, you are ready for a nursing home. I am not going to tell others.”

For people with YOD, the diagnosis is breaking the norms of an expected age-normative life course. Being and accepting a self-concept as elderly makes the diagnosis more comprehensible. One of the oldest, a woman, has told her family that she has got memory problems and received an understanding remark: “Yes, you are old.” Her problems are seen as normal aspects of ageing. A man said:

Officially, I am probably ill but feeling very well, and if I am not a burden, and all is OK, it is not a disaster. I am not young, shall not impress anybody, not make a fantastic career—in reality, what matters now is to manage everyday life. And I do that, I think, but lately, I do experience that it is harder to orientate myself when I am in town. Then we must take what comes. There is not much to do about it.

We see how his acceptance of his stage in life as an elderly man has subdued his ambitions for life and softened the impact of the disease.

### Social retraction

An accepting and supporting network is very important for their adaptation to the disease. A man underlined this: “I have a large network. They know I am a little bit outdated, but nobody cares anyway. So, we are still having very good times. I live as everything is OK.” He also spoke about a good relationship to his daughter and grandchildren. Nearly all the informants talked about increasing *social retraction* caused by their failing memory. Some already presented their retraction in the first interview, while others were very socially active and satisfied. In later interviews, they may remark that some social activities have become too demanding. Some informants are, as mentioned, forced into social retraction by being expelled from their work, and one informant said:

I feel very stable, I think. I find it OK. But it is sometimes boring. I had two jobs in many years. Suddenly I have nothing. That is what bothers me, but I just must do it. There is no alternative. I just have to stay with it.

One informant described in detail how her memory problems have caused her social retraction; she went from being a very social person to becoming isolated and lonely. She told how she stopped attending family gatherings, because she was no longer able to participate in party conversations:

I did not remember who had birthdays, when we were together the last time, and which grandmother was in the hospital … All those things you normally remember. It made such awkward situations, it was sad. It made me uncomfortable in social situations … I do not trust other people any longer.

Small talk is based on shared information and references, to names, places, participants and events. Not being able to recall makes the person a social “dropout”. To protect herself and not disclose her serious incapacity, she retracted. She said that she has broken off contact with “absolutely all old friends”. Faces, mimics and voices all give cues to get the meaning of words in conversations:

As a human being, you read others. I hate talking on the phone. Then I do not see the person, and it gets worse and worse. I do not see the eyes, the face, and the smile. So, then there are even more mistakes than when I have the person before me.

Body language gives important cues to the meaning of the conversation.

Two women have broken out of marriages or a partnership after getting the diagnosis but before the first interview. One of them told us in the second interview how her husband was not able to accept that she no longer was the person he knew and as he wanted her. She is now moving out to a new private flat and calls this stage of her life a “break-up stage”—both of marriage and of home. She is scared:

“It is very difficult. Sometimes I cry a lot. Life is changing, but now I fear for the future. How will I manage on my own, will I be able to handle the so-called ‘normal life’?”

The other woman who recently broke out of her partnership said that she became so irritated with her partner that she asked him to move out. To both of these women, coping with dementia seems easier when living alone.

The children are usually in the background in these stories. The informants who have children living far away see them on shorter visits, and they are not involved in the informant’s everyday life. Two women told how their daughters complained and even got angry when their mother made mistakes. One daughter became more understanding when she was informed of the diagnosis; the other daughter for a long time did not believe it. The three people with children living in the same town reported that the relationship is “good”. However, far more important in their narratives now are relationships with friends, people at (former) work, people in the neighbourhood or residents at the nursing home—all people they have been on a par with.

### Existential anxiety

One of the informants gave the most detailed description of anxiety and despair. She spoke about her new life alone: “I have got a serious morning anxiety.” After having “broken out” of marriage and moved to a new flat, life has become frightening:

I think it is difficult to start in the morning. I am scared. I just want to hide under my blanket. Not because I am lazy, I have never been lazy, but because I am terribly scared. I am scared for meeting a new day. You know, challenges, misunderstandings, things I do not master any longer.

The expanding experience of losing control and having little mastering capacity left evokes anxiety. The interviewer asked: “The anxiety and unrest you feel in body and soul, why is that so?” The interviewee answered: “Because I am losing control. I am no longer able to differentiate between danger and normality.” She panics when the doorbell rings, trusts no one since she fails to recognize her new neighbours and her home helpers: “It is terrible. I think it is so often that I must say ‘sorry’.” She has had to signal that something is “wrong” with her, and has informed everyone that she has “brain damage”. She feels that her neighbours are avoiding her. Her balcony is her retreat place. Even housework becomes uncontrollable. Every task takes much more effort, she does not remember what she intended to do, or when she performed the task the last time—like cleaning the floor. Her existence has been shrinking during the period of the past 2 years: “I do not enjoy and benefit by things as I did before.” She is exhausted and longs to be comforted and looked after. Her means to escape is alcohol: “Then I shut it out.”

### Revival of the self

Three people—two women and a man—have moved into a nursing home during the interview period. Going to live in a nursing home is a radical shift in the life situation. Only one informant described in detail what the new life means to her emotionally, cognitively and socially. She told us what a transition to a nursing home—at best—*can* mean for a person with dementia. In the third interview, she summarized the great change:

“I was so terribly scared by everything—even normal human situations. No, I am no longer scared—and it is a relief to avoid anxiety, and be so exhausted.”

Her life has improved dramatically. She no longer touches alcohol “because I am fine. I feel safe. I can even sit calm and knit, as I have not done for a long time. I feel it is quiet, even if the elderly here fuss a lot!” She has taken precautions to avoid her former feelings of loneliness and despair:

“I avoid sitting in my room too much, even if it is cosy, because I am so afraid of the old feelings. They still are there, in my heart, and I do not want to feel them anymore.”

She finds all her new neighbours nice and appreciates that “there is always someone to talk to”. Listening to other people around is now relaxing: “I often just sit in the living room, hear somebody around, the usual sounds, and I am pleased and can sit and look at TV programmes.” She summarized the most important differences between her former life and now: “Nobody here says that I have to control myself. Luckily. For the whole world outside does that.” Even her brain has relaxed: “Now my brain is working.” Her new vitality has even caused her to smarten up, present herself with her former image, to the annoyance of her former partner:

“He says: ‘No person with dementia can dress up.’ I say, ‘This is rubbish.’ I was so scared that I did not even think of being smart. But now I enjoy looking good.”

The institution is for her *peace*: “Here they do not repeat and repeat and always correct me, as they do outside.” Living in a place where they accept her, support her and are kind to her, *with* her dementia and her shortcomings, brings out her former self. She has now gone from passivity to activities. She is a gardener, as she used to be in her former life, takes photos and arranges exhibitions, and assists in housework: “It is important to be useful.” She is now “training and training” on her computer: “[I] will recall as much as possible of my former abilities.” Her remarks reveal very determined coping efforts. She even thinks that she remembers more “because I am calm inside me, I am more ‘gathered’ now. Now my anxiety does not take such a great place.” The core of her transformation of her experiences of living with dementia is the opportunity to be herself *with* the dementia, at the nursing home:

“It is so essential to be myself, for in my life outside I used so much energy to try, in a way, to appear as normal as possible. It always failed.”

Her fourth interview is a story about the remarkable revival of her former self, with new energy, vitality and lust for life. She is reconnecting to her identity, revitalizing old interests and knowledge and regaining meaning in life—with dementia.

The other woman who moved to a nursing home also greatly praised the change. Her narratives are short, mostly matter-of-fact stories. But she summarized the situation in these words: “Now I know that they are around me 24 hours. I call them angels, those working here. I am tremendously glad.” The man finds living at a nursing home tolerable but boring, with less freedom: “It is very nice here. Unproblematic. You rise up, wash your clothes, get food four times a day. But you do not have freedom any longer.” He feels he is stuck there: “The waking hours—17 hours a day—it is an awful lot of hours at this place, if you have nothing else to do.” The move—together with losing his driving certificate—means for him an emptier life. To him, living in nature, seeing birds and animals outside his house, was a meaningful life. In contrast to the informant who moved to a place where she found that her talents were supported, seen and revitalized, this man has lost the most existential meaningful aspects of his life.

### Worse and worse

A woman, still living at home at the last interview, mentioned her problems—apart from memory problems—as passivity, loss of energy and spirit, and lack of lust for life: “I get tired by just being awake.” Also, the future seems lost: “There is no future any more. I have been transformed to a person frightened about the future—taking the problems beforehand.” She underlined that she does not want to become “an empty shell”. She asked:

What is the point of prolonging life? Life is a hell, both to me and others. So, I think it is my choice. Now it [the dementia] shall just take its course. I have felt that I am remarkably worse. People do not feel it, but I feel it. So, I isolate myself much more.

She has ended all medication, and the contact with the home nurses and the dementia team. She did not “see the point”. Life has lost its meaning: “Do you know what is the worst? It is to get the days to pass, to finish one day after another. It is a slow torment.”

When the woman living in a nursing home was visited the last time, she was still very satisfied with her living conditions, but now she differentiates more between her outer life and the progression of the disease: Her first sentence was: “Here at the nursing home, I am fine. It is nice people here, and safe. But when it comes to myself, it is ups and downs.” Even if she still feels more emotionally calm, she easily gets uncontrolled, both in sorrow and joy. She is alarmed that her writing has deteriorated. The word “shame” returns, and she wants to reduce writing as much as possible—also to those she is fond of. Avoiding shame has returned as a strategy and a feeling: “I am not ashamed here, luckily, but outside if I do something. It is so easy to become ashamed. Then you get sorry, and feel stupid, when you have not been that. I have never been stupid before.” Her main self has been to be competent, to feel and be seen as stupid is a blow to her “master self”. She is performing most of her former activities, also complicated tasks, like paying her bills and knitting. But she no longer wants to mingle with co-residents in the common living room. She has found a semi-social way of being alone and social at the same time. She sits in her room with an open door, listening to others being there. This is her strategy to feel safe, surrounded by kind people, and to avoid the panic of aloneness and lack of control. She appreciates her quiet life: “I am happy just to sit and look at people. Too much fuss is awful.” She foresees further deterioration, losing abilities to perform her most meaningful and valued activities like knitting. Her horror image is just to be sitting in a chair, without any abilities and aspects of identity left:

“Then they have to bury me in the garden here.” Her last words are: “If I had dressed like a clown, maybe I would have passed.”

### Health personnel as background

The personnel from the health services that the people with YOD in this study have met play a minor part in the informants’ stories, in spite of receiving services of different kind, as shown in Table I. They figure as an undifferentiated and indifferent “background”. They are mostly anonymous, without personal characteristics and relations. When the informants are living at home, the health care personnel are experienced as an impersonal stream of people. They may be nice and efficient but seem to be insignificant in the stories. As the dementia disorder progresses, the nurses may come one or two times a day for specific tasks, like taking care of medication. One man gave the personnel a more significant place in his story:

The one who visited me this morning is one of my favourites. We are sitting and having a small talk, and that is very so … very bright in my day, those visits. It is the highlight of the day when they drop by.

For the three informants having support contacts, these have an important place in their stories.

They accompany them to valued activities, help them when they fail, and fill a void when the social network shrinks: “She is like a friend to me.” They flexibly assist where and when they are most needed. As mentioned, the two women who moved to a nursing home greatly appreciated the personnel as kind, understanding and supportive both in their daily life and to themselves. On the other hand, the man who moved to a nursing home found his days there boring, lacking meaning.

## Discussion

The study has shown that people with YOD are able to tell how they experience life with progressing dementia, even at stages when they are greatly affected by problems with short-term memory. This is in line with some exceptional books written by people with dementia (de Baggio, 2002, 2003; Taylor, 2007) as well as reports and recorded stories (Rose, 2003; Voris, Shabahangi, & Fox, 2009). Some people, like one of the informants, do have a large vocabulary to describe feelings, cognitive changes and social interactions, and can show how these domains are interwoven. Even if words disappear, much of a rich vocabulary can be preserved for a long time, and people with dementia give very valuable narratives of “Alzheimer’s from the inside” (Rose, 2003). Since our longitudinal study started shortly after (close to 6 months) the diagnosis was made, it included people with rather well-preserved verbal abilities. People have their own sociolect and dialect, as well as a personal tone—what Greenwood (Power, 2014) calls “the word palette”, which is the communicative fundament also when dementia sets in. Power (2014) has emphasized how words, signs, body language, mimicry and metaphors carry meaning. Studies (Arons, Krabbe, Schölzel-Dorenbos, van Der Wilt, & Rikkert, 2013; Sands, Ferreira, Stewart, Brod, & Yaffe, 2004) show that information about the quality of life of people with dementia by proxy (family members, healthcare personnel) deviates from their own experiences.

The main overriding theme in the informants’ stories about living with dementia over time is *changes of identity over time*. The narratives revolve around how to preserve one’s identity during time, to experience a lust for life and vitality, and to participate in human interactions that validate and support one’s identity.

In the initial stages, the increasing memory difficulties are seen mainly as an everyday problem which they try to cope with by different measures, such as taking notes and using Post-its. Gradually—sometimes slowly—the memory problems interfere more annoyingly with their daily life and social relations.

There is a close tie between identity and connectedness (Power, 2014). If possible, to preserve normal relationships, the informants avoid presenting themselves to others as carrying the diagnosis of dementia. They want to escape the stigma of the disease and the “worst case images” it evokes. As they mention, then the situation gets worse, for others as well as for themselves. The stigma of dementia is well documented by studies (Behuniak, 2011) and by reports from people with dementia (Aquilina & Hughes, 2006; Taylor, 2007), that tell how people immediately change their communication and relation when they are informed about the diagnosis (Simpson & Simpson, 1999). Metaphors often used about people with dementia like “half-empty”, fading away”, “no longer there” or “the living dead” (Behuniak, 2011) underline the dehumanization of those with the condition. The biomedical model presents reactions as symptoms of the disease and stress deficits (Power, 2014). Van Gorp and Vercruysse (2012) analysed how the media tend to reinforce the stigmatization of dementia as one of the most dreaded diseases in western society. People with dementia become “the demented other” (Sabat, Johnson, Swarbrick, & Keady, 2011). To avoid becoming “the other”, the informants prefer to present themselves as a person with a memory problem. This is a more usual and accepted disability, especially for elderly people.

In this way, the informants seem remarkably able to cope with the disease for a long time and to live a satisfying life, as they told us. After the initial shock of the diagnosis (Aminzadeh, Byszewski, Molnar, & Eisner, 2007), which some reported, they seem to have adapted rather well to the situation. In the early phases of the study, most of the informants find that their strategies for handling the life situation are sufficient for living a good life. They emphasized that they focus on the positive side of life. If they are able to live “a normal life”, which they are used to and value, and are connected to others in a life that is meaningful to them, most of them find that their quality of life is rather good. Many were still satisfied with life in the last interview we conducted.

Identity can be better preserved in the customary, well-known, everyday life, where people, surroundings, physical structures and material things are recognizable and support old habits (Arntzen, Holthe, & Jentoft, 2016). Living mostly like before also seems to protect against serious depression for our informants. For a long time, the lifestyle changes accompanying the development of the disease seem to make a greater impact on most of them than the immediate symptoms of the disease. They focus on changes like losing their job and their social and familial network, and moving to a new flat or to a nursing home.

An experience of continuity with their former self is essential, and the *continuity theory* of ageing of Atchley (1989) underlines the importance of *outer continuity* and *inner continuity*. These concepts refer to subjective experiences of the outer world and the inner self, and a feeling of continuity can be preserved also when the circumstances of life change. Familiarity fosters a sense of security, and we have demonstrated that feeling safe is important when dementia progresses. However, continuity may become too great and dysfunctional to maintain when old habits and ways of handling tasks no longer are possible to perform. As some informants reported, can continuity with partners and families be experienced as destructive when they are not able to tolerate the changes in personality that dementia implies. Studies have documented the great burden that family members experience caring for people with advanced dementia—“It is a torment.” Other people retract, the family carers become isolated, and they lose their own autonomy and personal life world ( Johannessen, Helvik, Engedal, & Thorsen, 2017).

There seems to be a tendency in the literature to overlook the conflicts and problems people with dementia may experience in family care. Support is not always given with warmth, love and concern. As one of the informants exemplified, her “new person” with dementia was no longer recognizable, acceptable or tolerated by those nearest to her. The “gap” between the former well-functioning person and the new, more disabled person will be especially great when people during the life cycle get dementia. The disease is unusual, unexpected and difficult to understand and to diagnose, even for medical personnel (Koedam et al., 2008). Our study has shown how people with dementia often found that social relationships with friends and partners became so complicated and destructive to their existential self that they preferred to break the contact. They life, become more isolated and get less identity support, falling into a negative spiral.

Living alone is not seen as a great disadvantage in our sample of single people, in contrast to research highlighting the importance of social support for quality of life, assistance and avoidance of depression. Three women—the two who divorced their husbands and a third who had divorced earlier—explicitly expressed the notion that living alone was an advantage. To manage alone is their way of life. At later stages, three people are no longer able to sustain existence on their own. The two women who moved into the nursing home greatly value the acceptance, support and validation (Feil, 2002) they get there. To them, the security and support they feel from the staff also strengthens them. They illustrate the great importance of a dedicated and accepting staff in good nursing homes. The third person, the man, found his days empty and did not feel existentially confirmed in these surroundings.

Our study underlines the importance of supporting the *personhood* for well-being among people with dementia, in line with the theory for dementia care presented by Kitwood and Bredin (1992). Further theoretical developments highlight the lesson: “Preserve identity, celebrate personhood and create meaning in the moment” (Power, 2014, p. 42). Many strategies for preserving identity focus on the life in the past—the individual’s life history. Further, in the dementia process, referring to the past becomes increasingly difficult, and less meaningful. One informant’s story shows how social interaction later becomes too demanding, and she prefers sitting and seeing others, knowing that they are there. Just *being* and *being safe* is more comforting to the self than doing and talking. Both breed failure. Well-being now becomes all-important.

Home nurses and home helpers are not mentioned by the informants as significant supporters; they belong to the background of everyday life. The findings seem to contrast with the fact that the people with YOD in this study receive health care services, as shown in Table I, but these helpers are not experienced as central to their existential life. The personnel give tolerable support, sometimes being nice, sometimes a nuisance—even scaring the informants when they do not recognize who has come to their home. As mentioned, one man finds the home nurses brighten his day; they talk and relate to him as an individual. However, the informants emphasized with warmth the support contacts, who were valued for their personalized and adapted contact “like a friend”, in accordance with the results in a study of support contacts (Johannessen, Engedal, & Thorsen, 2016). In addition, the Norwegian government’s *Dementia Plan 2020* recommends that people with dementia and their caregivers throughout the disease process should get a contact person in the health system—a person who should know the individual, and coordinate and plan the assistance. The individual should get varied support, outlined in an individualized care plan (HOD, 2012, 2015).

Moreover, public care in private homes and in nursing homes is based on standardized systems regulated by working schedules. Norway has many part-time health workers (Statistics Norway, 2011), which implies that clients meet many personnel, difficult to distinguish and remember for people with memory difficulties. The diagnosis of dementia, its signs and symptoms, place the person within the biomedical model, thus transforming the person into a patient. With the intention to change the culture of care, different concepts are introduced as person-centred care (Edvardsson, Winblad, & Sandman, 2008; Kitwood, 1997), person-directed care (Fox, Norton, Rashap, Angelelli, & Tellis-Nayak et al., 2005), relationship-centred care (Suchman, 2006) and authentic partnerships in care (Dupuis et al., 2012). It is vital that intentions of transforming public care to be person-orientated, supporting the identities of the persons with dementia, do not underrate the influences of the cultural and organizational/economic context (McCormack et al., 2002). To transform caring systems, it is crucial to direct strategies both at the culture and the physical/organizational structure, to give young people with dementia a better life living with the disease (McCormack & McCance, 2006; Power, 2010).

### Reflections on method

Performing a longitudinal in-depth study with interviews among people with dementia depends on trust between the informant and the interviewer (Kvale & Brinkmann, 2010). Trust has laid the foundation of this study and made possible continuing interviewing even when the dementia has deteriorated markedly, and the person has moved to a nursing home. The strength of this study is that the narrated stories are remarkable as unique documents about existential living over time with dementia. The stage of the dementia will certainly influence the narratives people may give, and the progressive disease restricts the sample and the time people can be followed. We claim that even if the sample is small, the themes have broader validity showing longitudinal experiences of single people living with YOD. Also, even though results of qualitative research designs cannot be generalized in a statistical sense, we argue that our results can be transferred to other contexts with people living with dementia. The findings may also contribute to a better understanding of how to contribute to the development and organization of services for people with dementia.

People in partnership will have other experiences. The findings might also be different if the participants with YOD were at the same stage in the dementia process and had the same diagnosis. However, we find that the variations in our sample of people with YOD offer new insight. To contribute to trustworthiness, quotations have been presented in the text. In addition, data were analysed and discussed between the authors (Lincoln & Guba, 1985).

## Conclusion

The main existential challenge for people with dementia, for a long period in the progress of the disease, is to preserve and protect their identity. The longitudinal study shows by the significant themes how the people react to handle the challenge. People with dementia experience and reflect on the changes brought by the disease, have full or partial insight for a long time, and try to master and control their life as the disease progresses. The voices of people with dementia should be listened to and included in planning public health services. Personalized care, like support contacts, should be used to assist people with YOD to preserve their identity in a normalized everyday life as far as possible.
